# Health-Related Factors in Rural and Urban Mexican Adolescents from the State of Jalisco: The HELENA-MEX Study

**DOI:** 10.3390/ijerph17238959

**Published:** 2020-12-02

**Authors:** María Rivera-Ochoa, Javier Brazo-Sayavera, Barbara Vizmanos-Lamotte, Asier Mañas, Juan Ricardo López-Taylor, Marcela González-Gross, Amelia Guadalupe-Grau

**Affiliations:** 1 ImFINE Research Group, Universidad Politécnica de Madrid, 28040 Madrid, Spain; mariariveraochoa@gmail.com (M.R.-O.); marcela.gonzalez.gross@upm.es (M.G.-G.); 2 Faculty of Sport Sciences, Universidad Pablo de Olavide, 41013 Seville, Spain; jbsayavera@cur.edu.uy; 3 Polo de Desarrollo Universitario EFISAL, Centro Universitario Regional Noreste, Universidad de la República, Rivera 40000, Uruguay; 4 Instituto de Nutrigenética y Nutrigenómica Traslacional, Centro Universitario de Ciencias de la Salud, Universidad de Guadalajara, Guadalajara 44430, Mexico; bvizmanos@yahoo.com.mx (B.V.-L.); taylor@cucs.udg.mx (J.R.L.-T.); 5 GENUD Toledo Research Group, University of Castilla-La Mancha, 45071 Toledo, Spain; asier.Manas@uclm.es; 6 CIBER of Frailty and Healthy Aging (CIBERFES), 28001 Madrid, Spain

**Keywords:** body composition, physical fitness, sedentary behavior, nutritional status

## Abstract

Mexico shows a high prevalence of obesity in children and adolescents. Geographical location and cultural environment could play a role in the promotion of healthy lifestyles in terms of physical activity (PA), sedentary behavior (SB) and nutrition. The purpose of this study was to assess rural and urban differences in body composition (BC), physical fitness (PF), PA and nutritional status of adolescents from the state of Jalisco (Mexico). The study involved 469 students aged 13–17 years (55.0% girls) from eight high schools. BC was analyzed by bioimpedance and PF by standardized field tests. Objective measurements of PA and SB were taken in a subsample (*n* = 240). Energy intake (EI) was calculated from two 24h recalls. Rural residents presented a higher prevalence of overweight, waist circumference, trunk fat mass, regional fat free mass and muscle handgrip strength (all *p* < 0.05, η2p < 0.06). Cardiorespiratory fitness was similar among participants, whereas urban adolescents showed higher muscle power, speed-agility and flexibility scores (all *p* < 0.05, η2p < 0.07). Overall lifestyle behavior in urban adolescents was more sedentary (*p* < 0.05, η2p = 0.11). EI was similar in both locations. In conclusion, rural Mexican adolescents presented a generally lower sedentary behavior and a lower fitness and fatness profile than their urban peers.

## 1. Introduction

Excess body fat is increasing at alarming rates across the globe [1]. According to World Health Organization (WHO) data, the prevalence of overweight and obesity among children and adolescents in Mexico is 34.4% and 13.5%, respectively [2]. The National Survey of Health and Nutrition (Encuesta Nacional de Salud y Nutricion in Spanish—ENSANUT) 2016 results have shown that those rates increase especially in Mexican adolescents living in rural depressed and low economical resources areas [3]. Therefore, it seems that geographical context needs to be taken into consideration when trying to expand the objective information on the causes and consequences of the obesogenic environment in Mexico. Compared with urban communities, Mexican rural communities have limited access to health care, lower economical and material resources and poorer public health promoting actions. In contrast, urbanization could be a factor that promotes sedentary behaviors, due to perceived safety of the environment that leads to spend less time in outdoor activities as well as an increasing use of technology for both professional and leisure purposes [4].

The relevance of following a healthy lifestyle during adolescence for current and future health has been widely reported in the scientific literature [5,6]. International initiatives like the HELENA study [7], developed in Europe, or the more recent one, Global Matrix [8], that integrates an important number of countries around the world, have pointed out the high level of physical inactivity among adolescents and the relevance of physical fitness and nutrition for adolescents’ health.

Recently, a study based on a global school-based survey highlighted that 81.0% of students (aged between 11 and 17 years old) do not meet the international guidelines on physical activity (PA) [9]. In Mexico, the 2018 report card on PA in children and adolescents informed about the low percentage of participants meeting the international guidelines, more notably in younger girls [10]. Only 60.5% of Mexican respondents between 15- and 19-years old follow the recommendation of 420 min of moderate-vigorous PA per week and 78.6% of them spend more than two hours in front of a screen [3]. Physical fitness (PF) is also considered an important indicator of good health [11]. Favorable associations have been reported linking cardiorespiratory and muscular fitness to cardiometabolic disease risk, adiposity, mental and bone health as well as cognition in adolescents [12,13]. In Mexico, it has been indicated that PF is a stronger correlate and better predictor of obesity than PA in schoolchildren [14]. However, the 2018 PA report card indicated that there is no sufficient data to grade the PF levels in Mexican adolescents [10]. Therefore, there is a lack of knowledge regarding this topic in this population group. Interestingly, different studies [15,16] suggest that the levels of PA and PF are different in urban and rural areas, as they are influenced by the environment context in which they live.

Regarding Mexican diet, it has been reported that 80% of Mexican adolescents consume sweet drinks and almost 60% eat snacks, sweets and non-healthy desserts. Among the Mexican adolescent population, 50% consume sweet cereals, 33% drink milk alternatives with added sugar and 20% eat processed meat, fast food and typical Mexican little snacks (“antojitos”). However, a low percentage consume fruits (39.2%) and vegetables (26.9%) [3].

Taking into consideration the present knowledge gaps related to a healthy lifestyle in Mexican youth and the importance of the place of residence as an environmental factor, the aim of this study is to report information about health related-factors such as body composition (BC), PF, PA, sedentary time (ST) and dietary intake (DI) of adolescents from the state of Jalisco (Mexico) comparing rural and urban participants.

## 2. Materials and Methods

### 2.1. Study Design

The Healthy Lifestyle in Mexico by Nutrition in Adolescence (HELENA) MEX study is a randomized multi-center cross sectional study based on the methodology used in the European HELENA study [17]. We examined adolescents’ lifestyle from rural areas (North Region of Jalisco) and urban areas (Metropolitan Area of Guadalajara). The methodological adaptation of the HELENA study to the Mexican population was carried out during September and October 2017. A group of collaborators were trained to collect data. In the urban area, the training was conducted between December 2017 and January 2018, whereas in the rural area it was carried out from March to April 2018. Data collection took place between January and June 2018. The study was approved by the Ethics, Research and Biosafety Committee of the University of Guadalajara, Mexico (CI-04717). Participants were invited to voluntarily participate in the study. A signed informed consent was obtained from all participants and their legal guardians prior to their participation in the study.

### 2.2. Study Sample

A random, stratified, multiple steps sampling was carried out. A list of all secondary and high schools of the North Region of Jalisco and the Metropolitan Area of Guadalajara was obtained from the Jalisco School Directory. Participating schools were randomly selected from the directory. Schools which were not willing to take part in the study or did not meet the criteria were excluded and the next school on the list was contacted. In most schools, one classroom was randomly selected for each age group (two classrooms per school in total). The study was explained and students within those two classes were asked to participate voluntarily. However, this protocol was not followed in one of the schools, since they asked to include different participants from all classes within the age range due to organizational purposes.

Taking the average rate of 30 students per class within the State of Jalisco, the protocol set up evaluating 240 adolescents of each area, 480 in total. A total of eight schools were chosen, four secondary schools and four high schools, in order to include adolescents between 13 and 17 years old. The selected classrooms in the secondary schools consisted on one group of students aged 13–14 and another group of participants aged 14–15. In the high schools, the selected groups were one group aged 15–16 and another group aged 16–17. However, when conducting the study in the urban area, some of the random classes chosen had more than 30 students each, and thus the total of participants analyzed was higher than expected.

### 2.3. Measures

#### 2.3.1. Physical Examination

A medical doctor performed an examination to determine the overall health status of each participant. The participants’ medical history was checked to know if there were any pathologies that would prevent them from participating in the study. Pubertal stage was self-reported with help of drawn images according to the method of Tanner and Whitehouse [18].

#### 2.3.2. Anthropometry and Body Composition

Anthropometric measurements were carried out in the morning prior to PF testing according to the procedures of the HELENA study [19]. Participants’ height was measured to the nearest 0.1 cm using a stadiometer (SECA 215, Hamburg, Germany). Body mass was assessed to the nearest 0.1 Kg using a precision digital scale (Inbody 120, BioSpace Co., Seoul, Korea), with participants dressed in light sports clothing and without shoes. Body mass index (BMI) and age- and sex-specific BMI z-scores according to the World Health Organization reference standards were calculated [20]. Adolescents were classified as thinness (T), normal-weight (NW), overweight (OW) or obese (O) considering the z-score obtained within the analysis (T: ≤ −2SD, NW: ≤ +1SD, OW: > +1SD and O > +2SD; +1SD is equivalent to BMI 25 kg/m^2^ at 19 years and +2SD is equivalent to BMI 30 kg/m^2^ at 19 years).

Waist and hip circumferences were measured in centimeters with an anthropometric tape to the nearest millimeter. The tape was placed horizontally just below the 12th rib and over the iliac crest. The hip circumference measurement was taken at the point yielding the maximum circumference over the buttocks, with the tape held in a horizontal plane. Waist-hip ratio (WHR) was calculated by dividing waist circumference (cm) by hip circumference (cm).

A bioelectric impedance analysis (BIA) device (BIA, Inbody 120, InBody Co., Ltd. Seoul, Korea) with tetrapolar-8-point-tactile electrodes was used to measure BC. Each device had two different frequencies (20 and 100 kHz) for impedance measurement in five body segments (the four extremities and the trunk) and has shown a good relative accuracy for assessing BC in children [21].

Fat mass (FM), and fat free mass (FFM) were normalized by height^2^. Studies have shown that FFM and weight scale with height to approximately the power of two, establishing an analytic framework for height-scaled indexes [22]. Therefore, fat mass index (FMI) [FM/height^2^] and fat free mass index (FFMI) [FFM/height^2^] have been reported. 

#### 2.3.3. Physical Fitness

All participants underwent a series of field-based physical health-related fitness component tests including muscular fitness, muscle power, speed/agility, flexibility and cardiorespiratory fitness (CRF).

To assess muscular fitness, we used the following tests:

- Handgrip test (upper-body). A digital dynamometer with adjustable grip was used (Smedley III, TTM, Tokyo, Japan). Adolescents squeezed the dynamometer gradually and continuously for at least 2 s, performing (alternately with both hands) the test twice using the optimal grip span. The optimal grip span was calculated using the equation proposed by Ruiz et al. [23]. Their arm was extended completely and making sure that the dynamometer was not in contact with any other part of their body. The best result per hand was recorded in kilograms (accurate to 0.1 kg). Handgrip measurements are expressed as the mean of the right and left side best values.
- Standing jump test (lower-body). Participants jumped as far as possible with their feet approximately shoulders width apart from a starting position immediately behind a line. Participants could use both arms to swing. The test was performed twice and the best result was recorded in centimeters.

To assess the muscle power the Squat Jump (SJ), the Counter Movement Jump (CMJ) and the Abalakov Jump (ABA) tests were used. Participants performed two series of jumps, one of each type and in the previous order, resulting on a total of six jumps. A Platform DIN-A2—BOSCO SYSTEM (BYOMEDIC, S.C.P., Barcelona, Spain) was used to perform the jumps. The jump height was recorded in centimeters and the best result obtained of each type was recorded.

- Squat Jump. Evaluation of muscle power. Participants performed a vertical jump without rebound movements starting from a half-squat position (both knees at 90 degrees) with trunk straight and both hands on hips. Hands were placed on their hips and participants were instructed not to use them during the jump.
- Counter Movement Jump. Evaluation of muscle power plus the elastic component of the muscles. On the platform in a standing position, with legs extended and both hands on their hips, adolescents performed a vertical jump with a prior fast counter movement reaching 90 degrees on flexion at knees. Hands were placed on their hips and participants were instructed not to use them during the jump.
- Abalakov Jump. Evaluation of muscle power plus elastic component of the muscles and coordination capacity. It is a CMJ where adolescents could use their arms and trunk free to allow the best movement coordination.

The speed-agility was assessed by the 4 × 10 m shuttle run test. Two parallel lines were drawn on the floor 10 m apart. Adolescents were asked to run as fast as possible from the starting line to the other line and returned to the starting line twice (4 × 10 m) crossing each line with both feet every time. When participants crossed any of the lines, they should pick up or exchange a sponge that had earlier been placed behind the lines. The stopwatch was stopped when participants crossed the end line with one foot. The test was performed twice and the best result was recorded to the nearest tenth of a second.

Flexibility was assessed by back-saver sit and reach test (BSSR). The test was performed with a standard box. Adolescents were in a seated position with one leg straight while the other one was bent at their knee. Bending the trunk, participants reached forward as far as possible. Adolescents had to push a small bar over the top of the box. The test was performed twice per leg with the best result recorded in centimeters. The average of the best distances reached by both legs was used in the analysis.

The endurance of CRF was assessed by the 20 m shuttle run test. Participants moved from one line to another, which were 20 m apart, and then reversed direction in accordance with a pace dictated by a sound signal. The initial speed was 8.5 km/h and increasing at 0.5 km per min intervals (1 min equals one stage). The test finished when the participant failed to reach the end lines concurrent with the audio signals on two consecutive occasions. Otherwise, the test ended when the participant stopped because of fatigue. The last completed stage or half-stage at which the participant dropped out was scored. Maximal oxygen consumption, VO_2_max (mL/kg/min) was estimated using the equation published by Léger [24]. The protocol carried out in each test is widely described in the Manual of Operation of The HELENA study [19].

#### 2.3.4. Physical Activity and Sedentary Time

Device-based PA and ST were assessed with validated tri-axial accelerometers (GT3X Actigraph, Pensacola, FL, USA). Participants were asked to always wear the accelerometer on the right hip with an adjustable elastic belt for 7 consecutive days during waking hours and to remove it only during water-based activities and while sleeping. Participants received the accelerometer together with written and verbal instructions and a practical demonstration on how to wear the accelerometer. Recommendations of previous studies were followed to collect the data [25]. Accelerometer data were recorded at a 30 Hz sampling rate and analyzed using the ActiLife software (light version 5.3.0, (ActiGraph, LLC, Pensacola, FL, USA). Data were reintegrated into 15-s epochs [26]. Non-wear time was defined as a minimum of 20 min with allowance of 1–2 min of counts below 100 counts [27].To classify PA and ST, the cut-points developed by Romazini et al. [28] were adopted using vector magnitude and 15-s epoch. To be included in the analysis, participants were required to reach a minimum of 10 h per day of wear time and at least 3 days a week, in which at least two days had to be on weekdays and one day on the weekend. In order to analyse the adherence of PA in the study with WHO recommendations, the accumulation of at least 60 min/day of moderate-vigorous physical activity was also assessed.

#### 2.3.5. Dietary Patterns

24-h recalls were employed to record the dietary intake of a weekend day and of a weekday. In order to have each participant’s food record, they were asked to recall the food they consumed on the last weekend day and to record it on a sheet of paper with the guidance of a trained nutritionist. They were also asked to do the same on their own but for the current day. On the following day, each student was interviewed individually with a nutritionist, who was visually assisted by a food photo album validated in adolescent Mexican population [29] to elaborate the 24-h recall.

The evaluations were carried out by a group of 6 nutritionists trained in the management of the photographic album [30]. Each nutritionist entered the data obtained in each 24-h recall in the online Nutricloud^®^ software (Nutricloud, Guadalajara, Mexico). With this software we obtained the average energy intake in kilocalories (kcal) and the intake from carbohydrates, lipids, saturated fatty acids and proteins in grams.

### 2.4. Data Analysis

Means, standard deviations and 95% confidence interval were calculated to describe anthropometry, BC, PF, PA, ST and DI and caloric expenditure. Daily caloric expenditure was calculated as the sum of the PA in kilocalories and the basal metabolic rate (BMR) estimated by BIA. Normality of continuous variables was verified by Kolmogorov-Smirnov tests and the homogeneity of variances by the Levene test. One-way analysis of covariance (ANCOVA), including sex and maturation as covariates, was done to test rural and urban groups differences in each variable. ANCOVAs were followed-up with Bonferroni- corrected post hoc tests. The effect sizes (ES) were calculated using the partial eta-squared (η2p) and their interpretation was based on the following criteria: 0.01 ≤ ES <0.06 small effects, 0.06 ≤ ES <0.14 moderate effects, ES ≥ 0.14 large effects [31]. The level of statistical significance was set at *p* < 0.05. All calculations were performed using IBM SPSS Statistics for Windows, version 25 (IBM Corp., Armonk, N.Y., USA).

## 3. Results

From a total of 508 participants who were asked to participate, 33 refrained from taking part and six were excluded for other reasons. Due to the lack of attendance to school during the data collection, not all participants in the study completed all the tests conducted. Therefore, the total number vary throughout different evaluations in the study. Regarding the accelerometry assessment, a subsample of 240 adolescents was evaluated due to limitations in the availability of accelerometers.

**Figure 1:**
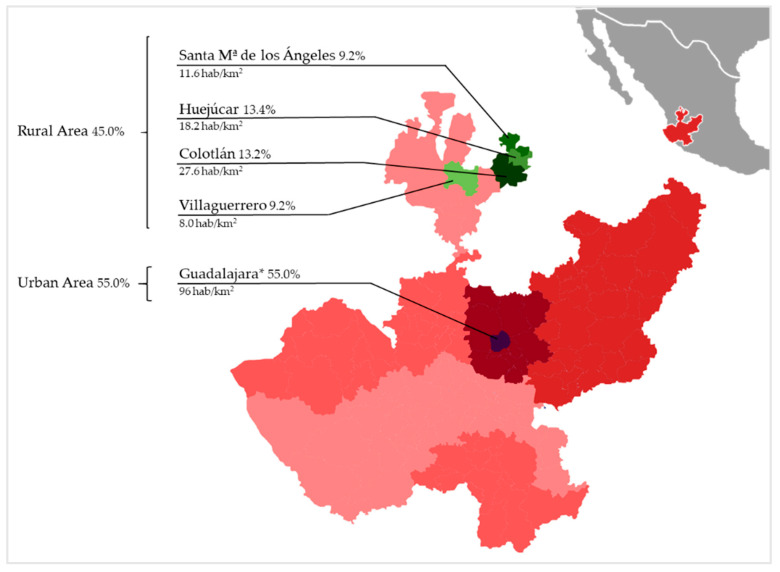
Percentage of adolescents evaluated and population density by area. * Metropolitan Area; green color = Rural Area; purple color = Urban Area.

Participants belonged to two different areas of the state of Jalisco: (1) urban population (Central Region) and (2) rural population (North Region). The Central Region, located in Guadalajara, concentrates 61.9% of the state’s population (5,023,785 inhabitants). The North Region is the least populated area of the state with only 1.1% of the population. In Guadalajara, four educational centers were chosen within the Metropolitan area. Likewise, four educational centers were chosen in different towns: Colotlán, Huejúcar, Santa María de los Ángeles and Villaguerrero. The population density of each town [32] and the participants evaluated by region (45.0% from the rural area and 55.0% from urban area) can be observed in Figure 1.

A total of 469 adolescents were recruited for the study (210 men, 44.8%). In the rural area, 37.9% out of 211 participants were males (males: 15.08 ± 1.33 years old; females: 15.25 ± 1.41 years old). In the urban area, 50.8% out of 258 participants were males (males: 15.32 ± 1.24 years old; females: 15.43 ± 0.96 years old).

### 3.1. Anthropometrics and Body Composition

Data for anthropometrics and BC are presented in Table 1. No significant differences were found in the height and weight of the participants living in the rural or urban area. The BMI (z-score calculated) and WHR were significantly lower in adolescents from urban areas even though the effect size was small, F(1445) = 4.53, *p* = 0.003, η2p = 0.02; and F(1462) = 9.07, *p* = 0.003, η2p = 0.02, respectively.

**Figure 2:**
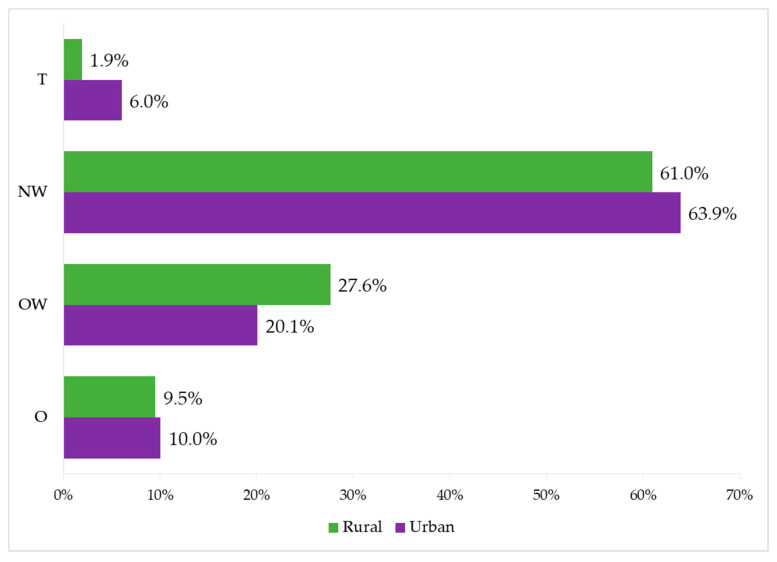
Classification of adolescents according to their BMI. T = Thinness; NW = Normal-weight; OW = Overweight; O = Obese.

The classification of participants according to their BMI is presented in Figure 2. Rural adolescents presented lower prevalence of T and O, while urban adolescents showed a lower prevalence of OW and a higher percentage of participants in NW.

No significant differences were found in the FM% and FFM% among adolescents in both areas. When the BC parameters where corrected by height, FMI in the trunk as well as FFMI in total body, arms and trunks were significantly higher in adolescents from rural areas in comparison to their peers from urban areas: F(1453) = 4.06, *p* = 0.045, η2p = 0.01; F(1453) = 7.04, *p* = 0.008, η2p = 0.02; F(1453) = 20.53, *p* < 0.001, η2p = 0.04; and F(1453) = 22.29, *p* < 0.001, η2p = 0.05, respectively.

Analyzing these results by sex, it was found that the WHR and FM in trunk are higher only in women from rural areas compared to women from urban areas: F(1255) = 8.95, *p* = 0.003, η2p = 0.03; and F(1254) = 4.12, *p* = 0.043, η2p = 0.02, respectively. Total FFMI was only higher in men from rural areas than those in urban areas, F(1197) = 5.85, *p* = 0.016, η2p = 0.03 (Table S1 in Supplementary Materials). Results show that all significant differences found between both groups in anthropometry and BC present a small effect size.

### 3.2. Physical Fitness

Regarding the muscular fitness of Mexican adolescents (Table 2), rural residents presented higher values in the handgrip test where F(1427) = 19.51, *p* < 0.001, η2p = 0.04, but no differences were found in the standing long jump. Urban residents obtained better results in muscle power (SJ test) and muscle power plus elastic component of the muscles and coordination capacity (ABA test), where F(1430) = 6.98, *p* = 0.009, η2p =0.02; and F(1432) = 5.64, *p* = 0.018, η2p =0.01, respectively. They also scored better in the assessment of speed-agility (4 × 10 m shuttle run test) and flexibility (BSSR test): F(1430) = 24.65, *p* < 0.001, η2p =0.05; and F(1432) = 31.29, *p* < 0.001, η2p =0.07, respectively. No significant differences were found in CRF, and VO_2_max estimated by Léger through 20 m shuttle run test. Results on all PF tests had a small effect size except for the flexibility test, where the effect size was found to be moderate.

Similar PF results were found when looking at sex differences. Women and men living in rural areas obtained better results in the handgrip test compared to their urban area counterparts but they obtained worse scores in the 40 × 10 m shuttle run and the BSSR test. Regarding the ABA test, results were higher for men from urban areas than from rural areas, F(1194) = 5.73, *p* = 0.018, η2p = 0.03 (S1).

### 3.3. Physical Activity and Sedentary Time

Significant differences were found in the values of daily PA between both groups with a moderate effect size (Table 3). 

Adolescents from the urban area spent a greater percentage of time in sedentary activities than those in the rural area: F(1236) = 28.72, *p* < 0.001, η2p = 0.11. Rural adolescents spent significantly more time than urban adolescents in light, moderate, vigorous and moderate-to-vigorous PA: F(1236) = 19.63, *p* < 0.001, η2p = 0.08; F(1236) = 15.43, *p* < 0.001, η2p = 0.06; and F(1236) = 16.40, *p* < 0.001, η2p = 0.06, respectively. The same results were observed when analyzing the results by sex (S1). 

**Figure 3:**
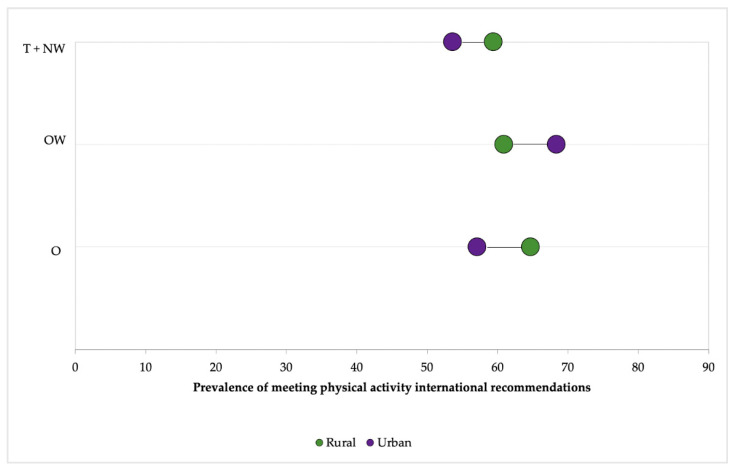
Prevalence of meeting physical activity international recommendations on PA for adolescents according to their BMI. T + NW = Thinness + Normal-weight; OW = Overweight; O = Obese.

Figure 3 shows a double stratification of living area and body composition categories. The thinness + normal weight (T + NW) category presented the lowest prevalence of recommended guidelines accomplishment (53.6%, urban area). OW urban adolescents shown the highest prevalence of recommended guidelines accomplishment (68.4%), while rural adolescents were more prevalent meeting PA international recommendations in the rest of categories (T+NW and O). Finally, adolescents included in the category T + NW presented the lowest gap between adolescents from the two analyzed areas (5.8%).

### 3.4. Dietary Intake and Energy Expenditure

No significant differences were found in total caloric intake or the intake of the different nutrients represented in Table 4 among adolescents in rural and urban areas. This was also the case when the sample was divided by sex (S1).

Daily caloric expenditure was higher in rural adolescents due to higher levels of PA: F(1235) = 10.81, *p* = 0.001, η2p = 0.04.

## 4. Discussion

The aim of the present study was to compare different health indicators among rural and urban adolescents in the state of Jalisco (Mexico). To the best of our knowledge, this is the first study performed in a developing country using the methodology from the European HELENA study that allowed us to gather information from a number of variables that are cornerstone for a healthy lifestyle in adolescents. Our results suggest that although rural Mexican adolescents are less sedentary than their urban peers (moderate effect size), they present a generally higher fatness profile (i.e FM in trunk, BMI and WHR, small effect size); and a lower fitness profile in terms of muscle power, agility-coordination and flexibility. Of note, cardiorespiratory fitness was similarly weak in both locations, and no differences were observed in dietary intake.

### 4.1. Anthropometrics and Body Composition

In line with what has been observed in the ENSANUT survey, we observed higher obesity and overweight scores, consistently shown by BMI z-scores, WHR and bioimpedance measurements in those adolescents living in rural areas, although it has to be noted that the effect size of this difference was small. Similarly to the results shown in the last ENSANUT survey, we find a prevalence of overweight and obesity higher than 30% in adolescents. This indicates the scope of this pandemic within the country and how serious it is in Mexico and Latin America [33]. The incidence of overweight and obesity in Mexico, as a whole, has not suffered significant increases between the National Health Surveys conducted in 2012 and 2016 except in the country’s rural areas where it has been exacerbated. A change has been observed in the evolution of overweight and obesity in regions with a higher socioeconomic level compared to more vulnerable regions and poorer households [34], which would explain the differences found in BC between rural and urban adolescents.

Urban adolescents participating in the current study showed lower indexes (BMI z-score and WHR) than those from rural areas. To this effect, there is a higher prevalence of overweight participants in the rural area. This observation was confirmed in our segmental body composition analysis, showing higher values of FM in the rural participant’s trunk. Central adiposity is one of the main determinants of cardiovascular (CV) disease [35], and our findings suggest that its role as a CV risk factor starts early in an alarming rate of Mexican adolescents. Accordingly, a previous study carried out in polish children observed the higher fat mass in girls and boys from a rural center and this resulted in their higher overall body weight compared to children from the city [36]. Levasseur analyzed a 10-year long panel data set from the Mexican Family Life Longitudinal survey (MxFLS). Interestingly, the author found that while anti-fat norms may particularly concern female, richer and urban students, pro-fat norms might persist among male, poorer and rural students [37]. Rural girls showed the highest trunk fat accumulation in the present study, although this was not associated to a less healthy dietary intake. Our observations reflect that the etiology of obesity is complex in the context of developing countries and more studies, especially those assessing sociocultural factors related to geographical location, are needed.

### 4.2. Physical Fitness

PF has been highlighted as a powerful marker of health in young people [11]. Physical fitness main components include cardiorespiratory fitness, muscular fitness, speed-agility and flexibility. Although PF is partly determined by genetic influence, several studies in different countries have examined its association with place of residence, as it can also be greatly influenced by environmental factors [38,39,40].

Regarding muscle fitness, rural residents from this study presented higher scores in the handgrip test. This can be in part explained by their higher level of FFMI in arms, as it has been extensively demonstrated in the literature the association between muscle mass and strength [41]. This is consistent with previous results that showed a positive correlation between BMI and handgrip strength in young Brazilian adults [42]. Moreover, high levels of muscular strength have been related to a more active lifestyle in Chilean children [43]. However, due to their higher weight, participants from rural areas might have more difficulties to move their body and this could be the reason for a lower level of muscle power, coordination capacity and speed in comparison to those from urban areas. Better infrastructure and social conditions in urban areas, having easier access to fitness and sports facilities [38] as well as having more opportunities to organize sport activities than young people in rural areas [39], have been highlighted as potential reasons for these geographical location differences. In addition, it has been observed that, in Ecuador, urban schools have more trained physical education teachers who are able to arrange a variety of sport activities and a more complete physical education program than rural schools, which count on less specialized teachers [40].

It should be noted that there are many contradictory results in literature indicating that better PF is more prevalent in adolescents in rural areas as is presented in the following information. In accordance with our results, measurements in Ecuador and Mexico [40,44] found that adolescents in urban areas had better results in muscle strength, speed agility, CRF and explosive power than their rural counterparts. Nonetheless, contrary results have been found in Spain [39], where young people living in rural areas had a higher score in those abilities. Our results show similar findings that a research carried out in Spain [39], where urban adolescents scored better in flexibility compared to their rural peers, which can be explained by an easier access to sports facilities.

Due to the inconsistency of results found in literature and the small effect size of these variables in the present investigation, clear conclusions cannot be drawn and attention must be paid when comparing results. As shown in other studies, these results could be influenced by the human development index [45]. For instance, there may be cultural differences among the studies, variations on the definition of urban and rural cultural environments as well as differences to evaluate physical abilities in each country.

A critical point to be considered is cardiovascular fitness. This dimension of PF refers to the overall capacity of the cardiovascular and respiratory systems and the ability to carry out prolonged strenuous exercise. A poor CRF (indicated as the maximal aerobic capacity or VO2max) has been traditionally defined as a risk factor for chronic disease and a recent systematic review shows the importance of this indicator in the future health of young people [46]. Regardless of place of residence, the adolescents from this study obtained VO2max scores around 30 mL/kg/min that classify as very weak. Therefore, increasing CRF values should be a priority in Mexican adolescents from the state of Jalisco.

### 4.3. Physical Activity and Sedentary Time

Regarding PA and ST, different evidence could justify the observed results. PA in developing countries like Mexico is associated with “active living”: active transportation, work and household tasks, especially in rural areas. Nevertheless, urban areas in Latin America are affected by crime, traffic insecurity and a lower availability of parks and open spaces, reducing the PA of residents in these areas [47]. Rural youth spend less time in front of the computer, video game or screens as well as sitting down [48], which could explain the lower values of sedentary time. Likewise, the active life in rural adolescence is associated with more physical labor as agriculture and handiwork or household chores [15].

Regarding the prevalence of minimum recommendations on PA accomplishment, the results found were similar to those reported by the latest National Health and Nutrition Survey 2016 in Mexico [49], where 39.5% (male: 30.1%; female: 48.8%) of adolescents did not meet the recommendations. When these data were related to the classification of adolescents as T, NW, OW and O, the results found were contradictory. In general, a higher percentage of rural participants follow the recommendations, which can be explained by active commuting to school, more common in rural Mexican areas [50]. However, a higher percentage of urban adolescents with OW practice 60 min of PA per day. This could be related to greater access to sports activities [48,51] and a greater support/monitoring of parents in the healthy behavior of their children in urban areas than in rural areas [52]. The smaller number of adolescents classified as OW and O or the differences of sex and age could distort the results found.

### 4.4. Dietary Intake

Dietary intake presented no statistical differences between groups probably due to the change in dietary habits around the world. Eating trends are changing and they are expected to keep changing in the upcoming decades. There is a general trend towards increases in the prevalence of obesity with economic development: fresh fruit and vegetables are becoming increasingly expensive in emerging economies while many processed foods are getting cheaper. In the last decades, fast food chains and poor-quality street food have become more accessible in the smallest and the furthest corners of the world. Over-consumption is rapidly moving to developing countries and rural areas [53]. According to Rivera et al. [33], higher prevalence of OW and O in Latin America might be explained by socioeconomic differences, and consumption of more industrially prepared foods than some other regions, such as sub-Saharan Africa and south Asia. In addition, the high socioeconomic level is more related to a healthy diet than the area of residence Other studies emphasize that the difference between the diet of rural and urban areas is in the lower consumption of junk food and more traditional food consumption [53]. The fact that there are no differences in the DI of both groups could be explained due to the increase of fast-food trends in rural areas of Mexico. To analyze the differences between rural and urban adolescents, we will have to take into account the socioeconomic status of each family and the advancement of fast food in Mexico and, therefore, analyze not only the macronutrient intake of rural and urban adolescents but also their eating patterns.

### 4.5. Strengths and Limitations

A strength of the present study was the strict standardization of the fieldwork by using a methodological of the HELENA study [17]. An additional advantage of the study was the use of accelerometry to measure ST and PA. Most studies carried out in Mexico evaluate these variables through questionnaires of PA [9,33,43,50,53]. As a limitation, availability in time and quantity of accelerometers decreased the sample of this variable. Only a subsample of 240 adolescents was evaluated and 66.25% of the sample was rural population. The daily activity of schools made difficult measuring all adolescents with field tests. We had to adapt to the normal operation of each school, and it was not possible to get 100% of evaluations on every single field test.

Dietary intake is difficult to measure. Thus, the 24-h recalls and food records are subjective estimates [53]. There is an inherent bias related to self-report in this type of assessment. However, in this study we used a food photo album as well as a nutritional software created specifically for Mexican population. Furthermore, trained nutritionists conducted these tests in order to reduce this bias.

## 5. Conclusions

Results from our study showed that rural Mexican adolescents accumulate less ST and more time in all intensities of PA throughout the day. Urban adolescents have a healthier BC than their rural counterparts. They obtain lower values of FM in trunk, BMI and WHR and higher scores in the explosive-strength, speed-agility and flexibility tests. However, rural adolescents presented higher values in FFM in arms and trunk, which could influence the higher result in the hand grip test. Except for PA, the differences found have a small effect size. The differences in PA did not explain differences in BC between urban and rural adolescents and we found no differences in DI that could help to clarify it. Additional studies are needed to explain if the origin of the nutrients and the eating patterns of rural and urban adolescents can demonstrate the differences found in BC and PA. Expanding knowledge about health-related factors could help the design of better public health strategies.

This evidence is important given that it can help guide future public health initiatives aimed at battling childhood obesity and sedentarism in Mexico. Research involving larger, more representative population samples is required to further explore these associations in Mexican children. The present findings may serve as an urgent call for action for Mexican and international public policy making, with special focus on anti-obesity programs in rural areas of residence.

## Figures and Tables

**Table 1:** Descriptive statistics and comparisons of anthropometry and body composition in rural and urban adolescents.

Variables	Rural		Urban	
	*n*	M ± SD	*n*	M ± SD
Height (cm)	211	162.42 ± 0.47	258	163.09 ± 0.42
Weight (kg)	210	59.73 ± 0.91	249	57.83 ± 0.83
BMI for age (z-score)	209	0.59 ± 0.09	247	0.23 ± 0.08 *
WHR	210	0.81 ± 0.00	256	0.80 ± 0.00 *
Fat mass (%)	209	27.15 ± 0.58	248	26.00 ± 0.53
FMI (FM/height^2^)	209	6.39 ± 0.21	248	5.99 ± 0.20
FMI arms	209	0.88 ± 0.03	242	0.80 ± 0.03
FMI trunk	209	3.21 ± 0.12	248	2.89 ± 0.11 *
FMI legs	209	1.90 ± 0.06	248	1.86 ± 0.05
Fat Free Mass (%)	209	72.85 ± 0.58	248	74.00 ± 0.53
FFMI (FFM/height^2^)	209	16.14 ± 0.13	248	15.67 ± 0.12 *
FFMI arms	209	1.64 ± 0.02	248	1.50 ± 0.02 *
FFMI trunk	209	7.31 ± 0.06	248	6.9 ± 0.06 *
FFMI legs	209	1.90 ± 0.06	248	1.86 ± 0.05

Note: BMI = Body Mass Index; WHR = Waist-Hip Ratio; FMI = Fat Mass Index; FFMI = Fat Free Mass Index. * Significant differences between rural vs. urban areas (*p* < 0.05).

**Table 2:** Descriptive statistics and results of physical fitness ANCOVAs in rural and urban adolescents.

Variables	Rural		Urban	
	*n*	M ± SD	*n*	M ± SD
Handgrip (kg) ^1^	204	28.73 ± 0.42	227	26.15 ± 0.40 *
Standing long jump (cm)	207	144.24 ± 1.74	227	146.38 ± 1.66
SJ (cm)	207	20.42 ± 0.36	227	21.75 ± 0.34 *
CMJ (cm)	207	21.81 ± 0.36	227	21.29 ± 0.34
ABA (cm)	208	25.27 ± 0.42	228	26.68 ± 0.40 *
VO_2_max (ml/kg/min)	204	29.84 ± 0.41	185	30.13 ± 0.43
4 × 10 m shuttle run test (sec)	207	13.35 ± 0.09	227	12.75 ± 0.08 *
BSSR (cm) ^1^	208	23.54 ± 0.51	228	27.53 ± 0.49 *

Note: SJ = Squat Jump; CMJ = Counter Movement Jump; ABA = Abalakov Jump; VO_2_max = maximal oxygen consumption; BSSR = Back-saber sit and reach. ^1^ Handgrip and BSSR are expressed as mean of right and left side. * Significant differences between rural vs. urban areas (*p* < 0.05).

**Table 3:** Descriptive statistics and results of physical activity (PA) and sedentary time (ST) ANCOVAs in rural and urban adolescents.

Variables	Rural		Urban	
	*n*	M ± SD	*n*	M ± SD
ST (%)	159	68.24 ± 0.52	81	73.14 ± 0.74 *
Light PA (%)	159	21.49 ± 0.35	81	18.77 ± 0.49 *
Moderate PA (%)	159	6.01 ± 0.16	81	4.94 ± 0.22 *
Vigorous PA (%)	159	4.26 ± 0.16	81	3.15 ± 0.22 *
MVPA (%)	159	10.28 ± 0.26	81	8.09 ± 0.37 *
MVPA (min/day)	159	77.05 ± 2.12	81	61.97 ± 2.99 *

Note: MVPA = Moderate to Vigorous PA. * Significant differences between rural vs. urban areas (*p* < 0.05).

**Table 4:** Descriptive statistics and comparisons of dietary intake (DI) and caloric expenditure in rural and urban adolescents.

Variables	Rural		Urban	
	*n*	M ± SD	*n*	M ± SD
Carbohydrates (g)	205	266.36 ± 6.70	227	264.15 ± 6.36
Lipids (g)	205	70.64 ± 2.38	229	66.04 ± 2.25
Saturated fatty acids (g)	205	22.28 ± 0.84	229	24.12 ± 0.80
Proteins (g)	200	81.00 ± 2.39	226	80.75 ± 2.24
Caloric intake (kcal/day)	205	1993.67 ± 47.33	227	1949.06 ± 44.92
BMR (kcal/day)	209	1302.60 ± 10.25	248	1277.12 ± 9.39
PA (kcal/day)	159	399.06 ± 14.72	81	292.42 ± 20.78
BMR + PA (kcal/day)	159	1686.22 ± 24.90	80	1542.46 ± 35.38 *

Note: BMR = Basal Metabolic Rate; PA = Physical Activity. * Significant differences between rural vs. urban areas (*p* < 0.05).

## Supplementary Materials

The following are available online at https://www.mdpi.com/1660-4601/17/23/8959/s1, Table S1: Descriptive statistics and results ANCOVAs in rural and urban adolescents by sex.

## References

[1] Hejazi S., Dahinten V.S., Ratner P.A., Marshall S.K., Bagchi D. (2019). Developmental Trajectories of Weight Status in Childhood and Adolescence. Global Perspectives on Childhood Obesity.

[2] The Global Health Observatory Explore a World of Health Data. https://www.who.int/data/gho/data/indicators.

[3] Romero-Martinez M., Shamah-Levy T., Cuevas-Nasu L., Gomez-Humaran I.M., Gaona-Pineda E.B., Gomez-Acosta L.M., Rivera-Dommarco J.A., Hernandez-Avila M. (2017). Methodological design of the National Health and Nutrition Survey 2016. Salud. Publica. Mex..

[4] Aceves-Martins M., Llaurado E., Tarro L., Sola R., Giralt M. (2016). Obesity-promoting factors in Mexican children and adolescents: Challenges and opportunities. Glob. Health Action.

[5] Mintjens S., Menting M.D., Daams J.G., Van Poppel M.N.M., Roseboom T.J., Gemke R. (2018). Cardiorespiratory Fitness in Childhood and Adolescence Affects Future Cardiovascular Risk Factors: A Systematic Review of Longitudinal Studies. Sports Med..

[6] Hallal P.C., Victoria C.G., Azevedo M.R., Wells J.C.K. (2006). Adolescent Physical Activity and Health. A Systematic Review. Sports Med..

[7] De Henauw S., Gottrand F., De Bourdeaudhuij I., Gonzalez-Gross M., Leclercq C., Kafatos A., Molnar D., Marcos A., Castillo M., Dallongeville J. (2007). Nutritional status and lifestyles of adolescents from a public health perspective. The HELENA Project—Healthy Lifestyle in Europe by Nutrition in Adolescence. J. Public Health.

[8] Aubert S., Barnes J.D., Abdeta C., Abi Nader P., Adeniyi A.F., Aguilar-Farias N., Andrade Tenesaca D.S., Bhawra J., Brazo-Sayavera J., Cardon G. (2018). Global Matrix 3.0 Physical Activity Report Card Grades for Children and Youth: Results and Analysis From 49 Countries. J. Phys. Act. Health.

[9] Guthold R., Stevens G.A., Riley L.M., Bull F.C. (2020). Global trends in insufficient physical activity among adolescents: A pooled analysis of 298 population-based surveys with 1·6 million participants. Lancet Child. Adolesc. Health.

[10] Galaviz K.I., Garcia G.A., Gaytan-Gonzalez A., Gonzalez-Casanova I., Gonzalez Villalobos M.F., Jauregui A., Jauregui Ulloa E., Medina C., Pacheco Miranda Y.S., Perez Rodriguez M. (2018). Results from Mexico’s 2018 Report Card on Physical Activity for Children and Youth. J. Phys. Act. Health.

[11] Ortega F.B., Ruiz J.R., Castillo M.J., Sjostrom M. (2008). Physical fitness in childhood and adolescence: A powerful marker of health. Int. J. Obes. (Lond.).

[12] Ruiz J.R., Castro-Pinero J., Artero E.G., Ortega F.B., Sjostrom M., Suni J., Castillo M.J. (2009). Predictive validity of health-related fitness in youth: A systematic review. Br. J. Sports Med..

[13] Smith J.J., Eather N., Morgan P.J., Plotnikoff R.C., Faigenbaum A.D., Lubans D.R. (2014). The health benefits of muscular fitness for children and adolescents: A systematic review and meta-analysis. Sports Med..

[14] Galaviz K.I., Tremblay M.S., Colley R., Jauregui E., Lopez y Taylor J., Janssen I. (2012). Associations between physical activity, cardiorespiratory fitness, and obesity in Mexican children. Salud. Publica. Mex..

[15] Regis M.F., Oliveira L.M., Santos A.R., Leonidio A.D., Diniz P.R., Freitas C.M. (2016). Urban versus rural lifestyle in adolescents: Associations between environment, physical activity levels and sedentary behavior. Einstein (Sao Paulo).

[16] Glaner M.F. (2005). Aptidão física relacionada à saúde de adolescentes rurais e urbanos em relação a critérios de referência. Rev. Bras. Educ. Fís. Esp..

[17] Moreno L.A., De Henauw S., Gonzalez-Gross M., Kersting M., Molnar D., Gottrand F., Barrios L., Sjostrom M., Manios Y., Gilbert C.C. (2008). Design and implementation of the Healthy Lifestyle in Europe by Nutrition in Adolescence Cross-Sectional Study. Int. J. Obes. (Lond.).

[18] Tanner J.M., Whitehouse R.H. (1976). Clinical longitudinal standards for height, weight, height velocity, weight velocity, and stages of puberty. Arch. Dis. Child..

[19] Gonzalez-Gross M., De Henauw S., Gottrand F., Gilbert C.C., Moreno L.A. (2013). Manual of Operation. The HELENA Study.

[20] De Onis M., Onyango A.W., Borghi E., Siyam A., Nishida C., Siekmann J. (2007). Development of a WHO growth reference for school-aged children and adolescents. Bull. World Health Organ..

[21] Khan S., Xanthakos S.A., Hornung L., Arce-Clachar C., Siegel R., Kalkwarf H.J. (2020). Relative Accuracy of Bioelectrical Impedance Analysis for Assessing Body Composition in Children with Severe Obesity. J. Pediatr. Gastroenterol. Nutr..

[22] Heymsfield S.B., Gallagher D., Mayer L., Beetsch J., Pietrobelli A. (2007). Scaling of human body composition to stature: New insights into body mass index. Am. J. Clin. Nutr..

[23] Ruiz J.R., Espana-Romero V., Ortega F.B., Sjostrom M., Castillo M.J., Gutierrez A. (2006). Hand span influences optimal grip span in male and female teenagers. J. Hand Surg Am..

[24] Leger L.A., Mercier D., Gadoury C., Lambert J. (1988). The multistage 20 metre shuttle run test for aerobic fitness. J. Sports Sci.

[25] Migueles J.H., Cadenas-Sanchez C., Ekelund U., Delisle Nystrom C., Mora-Gonzalez J., Lof M., Labayen I., Ruiz J.R., Ortega F.B. (2017). Accelerometer Data Collection and Processing Criteria to Assess Physical Activity and Other Outcomes: A Systematic Review and Practical Considerations. Sports Med..

[26] Garaulet M., Ortega F.B., Ruiz J.R., Rey-Lopez J.P., Beghin L., Manios Y., Cuenca-Garcia M., Plada M., Diethelm K., Kafatos A. (2011). Short sleep duration is associated with increased obesity markers in European adolescents: Effect of physical activity and dietary habits. The HELENA study. Int. J. Obes. (Lond.).

[27] Troiano R.P., Berrigan D., Dodd K.W., Masse L.C., Tilert T., McDowell M. (2007). Physical activity in the United States measured by accelerometer. Med. Sci. Sports Exerc..

[28] Romanzini M., Petroski E.L., Ohara D., Dourado A.C., Reichert F.F. (2014). Calibration of ActiGraph GT3X, Actical and RT3 accelerometers in adolescents. Eur. J. Sport Sci..

[29] Bernal-Orozco M.F., Vizmanos-Lamotte B., Rodriguez-Rocha N.P., Macedo-Ojeda G., Orozco-Valerio M., Roville-Sausse F., Leon-Estrada S., Marquez-Sandoval F., Fernandez-Ballart J.D. (2013). Validation of a Mexican food photograph album as a tool to visually estimate food amounts in adolescents. Br. J. Nutr..

[30] Vizmanos-Lamotte B., López-Uriarte P.J., Hunot-Alexander C., Bernal-Orozco M.F., Rodriguez-Rocha N.P., Macedo-Ojeda G. (2015). Álbum Fotográfico de Alimentos Mexicanos. Manual para la Estimación Visual de Cantidades.

[31] Cohen J. (1988). Statistical Power Analysis for the Behavioral Sciences.

[32] Encuesta Intercensal 2015. https://www.inegi.org.mx/programas/intercensal/2015/.

[33] Rivera J.Á., De Cossío T.G., Pedraza L.S., Aburto T.C., Sánchez T.G., Martorell R. (2014). Childhood and adolescent overweight and obesity in Latin America: A systematic review. Lancet Diabetes Endocrinol..

[34] Hernandez-Cordero S., Cuevas-Nasu L., Moran-Ruan M.C., Mendez-Gomez Humaran I., Avila-Arcos M.A., Rivera-Dommarco J.A. (2017). Overweight and obesity in Mexican children and adolescents during the last 25 years. Nutr. Diabetes.

[35] Chwalczynska A., Rutkowski T., Jedrzejewski G., Wojtowicz D., Sobiech K.A. (2018). The Comparison of the Body Composition of Children at the Early School Age from Urban and Rural Area in Southwestern Poland. Biomed. Res. Int..

[36] Lopes J., Grams S.T., Da Silva E.F., De Medeiros L.A., De Brito C.M.M., Yamaguti W.P. (2018). Reference equations for handgrip strength: Normative values in young adult and middle-aged subjects. Clin. Nutr..

[37] Garcia-Hermoso A., Cofre-Bolados C., Andrade-Schnettler R., Ceballos-Ceballos R., Fernández-Vergara O., Vegas-Heredia E., Ramíres-Vélez R., Izquierdo M. (2018). Normative reference values for handgrip strength in Chilean children at 8–12 years old using the empirical distribution and the Lambda, Mu, and sigma statistical methods. J. Strength Cond. Res..

[38] Gordon-Larsen P., Nelson M.C., Page P., Popkin B.M. (2006). Inequality in the built environment underlies key health disparities in physical activity and obesity. Pediatrics.

[39] Chillon P., Ortega F.B., Ferrando J.A., Casajus J.A. (2011). Physical fitness in rural and urban children and adolescents from Spain. J. Sci. Med. Sport.

[40] Andrade S., Ochoa-Avilés A., Lachat C., Escobar P., Verstraeten R., Van Camp J., Donoso S., Rojas R., Cardon G., Kolsteren P. (2014). Physical fitness among urban and rural Ecuadorian adolescents and its association with blood lipids: A cross sectional study. BMC Pediatrics.

[41] Pena Reyes M.E., Tan S.K., Malina R.M. (2003). Urban-rural contrasts in the physical fitness of school children in Oaxaca, Mexico. Am. J. Hum. Biol..

[42] Dumuid D., Maher C., Lewis L.K., Stanford T.E., Martin Fernandez J.A., Ratcliffe J., Katzmarzyk P.T., Barreira T.V., Chaput J.P., Fogelholm M. (2018). Human development index, children’s health-related quality of life and movement behaviors: A compositional data analysis. Qual. Life Res..

[43] Rodrigues A.N., Perez A.J., Carletti L., Bissoli N.S., Abreu G.R. (2006). Maximum oxygen uptake in adolescents as measured by cardiopulmonary exercise testing: A classification proposal. J. Pediatr. (Rio. J.).

[44] Day K. (2018). Physical Environment Correlates of Physical Activity in Developing Countries: A Review. J. Phys. Act. Health.

[45] Hermosillo-Gallardo M.E., Jago R., Sebire S.J. (2018). Association between urbanicity and physical activity in Mexican adolescents: The use of a composite urbanicity measure. PLoS ONE.

[46] Shamah-Levy T., Cuevas-Nasu L., Gaona-Pineda E.B., Gomez-Acosta L.M., Morales-Ruan M.D.C., Hernandez-Avila M., Rivera-Dommarco J.A. (2018). [Overweight and obesity in children and adolescents, 2016 Halfway National Health and Nutrition Survey update]. Salud. Publica. Mex..

[47] Jauregui A., Medina C., Salvo D., Barquera S., Rivera-Dommarco J.A. (2015). Active Commuting to School in Mexican Adolescents: Evidence from the Mexican National Nutrition and Health Survey. J. Phys. Act. Health.

[48] Chen C., Tsai L.T., Lin C.F., Huang C.C., Chang Y.T., Chen R.Y., Lyu S.Y. (2017). Factors influencing interest in recreational sports participation and its rural-urban disparity. PLoS ONE.

[49] Vancampfort D., Van Damme T., Firth J., Smith L., Stubbs B., Rosenbaum S., Hallgren M., Hagemann N., Koyanagi A. (2019). Correlates of physical activity among 142,118 adolescents aged 12-15years from 48 low- and middle-income countries. Prev. Med..

[50] Malik V.S., Willett W.C., Hu F.B. (2013). Global obesity: Trends, risk factors and policy implications. Nat. Rev. Endocrinol..

[51] Mayen A.L., Marques-Vidal P., Paccaud F., Bovet P., Stringhini S. (2014). Socioeconomic determinants of dietary patterns in low- and middle-income countries: A systematic review. Am. J. Clin. Nutr..

[52] Nzefa Dapi L., Nouedoui C., Janlert U., Håglin L. (2005). Adolescents’ food habits and nutritional status in urban and rural areas in Cameroon, Africa. Food Nutr. Res..

[53] Hermosillo-Gallardo M.E., Jago R., Sebire S.J. (2017). The Associations Between Urbanicity and Physical Activity and Sitting Time in Mexico. J. Phys. Act. Health.

