# Supplementary material for: Health-Related Factors in Rural and Urban Mexican Adolescents from the State of Jalisco: The HELENA-MEX Study
Source: Int J Environ Res Public Health. 2020 Dec 2;17(23):8959. doi: 10.3390/ijerph17238959 (PMC7729600; doi:10.3390/ijerph17238959)
Supplement: Supplementary file 1 [file ijerph-17-08959-s001.pdf]

Table S1. Descriptive statistics and results ANCOVAs in rural and urban adolescents by sex.

	Male				Female			
	Rural		Urban		Rural		Urban	
	n	M ± SD	n	M ± SD	n	M ± SD	n	M ± SD
**Anthropometry and BC**								
Height (cm)	78	167.98 ± 0.85	130	168.14 ± 0.66	133	162.42 ± 0.47	128	157.92 ± 0.51
Weight (kg)	78	64.20 ± 1.73	123	60.92 ± 1.37	132	56.55 ± 0.96	126	55.37 ± 0.98
BMI for age (z-score)	77	0.67 ± 0.16*	122	0.23 ± 0.12*	132	0.56 ± 0.10*	125	0.21 ± 0.10*
WHR	78	0.84 ± 0.01	130	0.83 ± 0.00	132	0.79 ± 0.00*	126	0.77 ± 0.00*
Fat mass (%)	78	20.61 ± 0.99	122	19.42 ± 0.79	131	32.46 ± 0.67	126	30.88 ± 0.68
FMI (FM/height^2)	78	4.92 ± 0.34	122	4.51 ± 0.27	131	7.62 ± 0.27	126	7.05 ± 0.27
FMI arms	78	0.60 ± 0.05	119	0.53 ± 0.04	131	1.11 ± 0.04	123	1.00 ± 0.05
FMI trunk	78	2.45 ± 0.19	122	2.15 ± 0.15	131	3.84 ± 0.14*	126	3.42 ± 0.15*
FMI legs	78	1.48 ± 0.09	122	1.41 ± 0.07	131	2.25 ± 0.07	126	2.18 ± 0.07
Fat Free Mass (%)	78	79.39 ± 0.99	122	80.58 ± 0.79	131	67.54 ± 0.67	126	69.12 ± 0.68
FMI (FFM/height^2)	78	17.68 ± 0.25*	122	16.90 ± 0.20*	131	14.98 ± 0.13	126	14.73 ± 0.13
FFMI arms	78	1.89 ± 0.04*	122	1.71 ± 0.03*	131	1.45 ± 0.02*	126	1.34 ± 0.02*
FFMI trunk	78	7.89 ± 0.12*	122	7.38 ± 0.09*	131	6.87 ± 0.07*	126	6.53 ± 0.07*
FFMI legs	78	1.48 ± 0.09	122	1.41 ± 0.07	131	2.25 ± 0.07	126	2.18 ± 0.07
**Physical Fitness**								
Handgrip (kg)[1]	74	34.44 ± 0.85*	120	30.63 ± 0.67*	130	24.15 ± 0.38*	107	22.73 ± 0.42*
Standing longjump (cm)	77	164.31 ± 3.36	120	169.91 ± 2.69	130	126.60 ± 1.72	107	126.98 ± 1.90
SJ (cm)	77	23.48 ± 0.69*	119	25.85 ± 0.56*	130	17.69 ± 0.36	108	18.34 ± 0.40
CMJ (cm)	77	25.34 ± 0.66	119	24.63 ± 0.53	130	18.79 ± 0.38	108	18.73 ± 0.42
ABA (cm)	77	29.32 ± 0.80*	120	31.78 ± 0.64*	131	21.65 ± 0.43	108	22.52 ± 0.48
VO_2max (ml/kg/min)	76	34.36 ± 0.92	92	35.17 ± 0.83	128	26.30 ± 0.29	93	26.32 ± 0.34
4x10m shuttle run test (sec)	76	12.46 ± 0.14*	120	11.65 ± 0.11*	131	15.12 ± 0.11*	107	13.67 ± 0.12*
BSSR (cm)[1]	77	21.19 ± 0.86*	120	24.56 ± 0.71*	131	25.43 ± 0.59*	108	30.20 ± 0.66*
**Physical Activity**								
SED (%)	52	62.51 ± 1.03*	43	69.67 ± 1.13*	107	71.81 ± 0.56*	38	74.84 ± 0.94*
Light PA (%)	52	24.52 ± 0.68*	43	20.50 ± 0.75*	107	19.59 ± 0.38*	38	17.98 ± 0.65*
Moderate PA (%)	52	7.05 ± 0.31*	43	5.60 ± 3.43*	107	5.37 ± 0.17*	38	4.59 ± 2.85*
Vigorous PA (%)	52	5.90 ± 0.33*	43	4.23 ± 0.36*	107	3.23 ± 0.16*	38	2.59 ± 0.26*
MVPA (%)	52	12.95 ± 0.55*	43	9.83 ± 0.60*	107	8.60 ± 0.27*	38	7.18 ± 0.46*
MVPA (min/day)	52	97.90 ± 4.35*	43	75.41 ± 4.78*	107	63.97 ± 2.19*	38	55.04 ± 3.69*
**Dietary Intake**								
Carbohydrates (gr)	76	285.96 ± 11.52	115	286.88 ± 9.36	129	249.22 ± 8.00	112	247.24 ± 8.60
Lipids (gr)	76	78.17 ± 4.11	117	73.05 ± 3.31	129	64.14 ± 2.81	112	62.09 ± 3.02
Saturated fatty acids (gr)	76	25.08 ± 1.45	117	27.02 ± 1.16	129	19.91 ± 1.01	112	21.93 ± 1.09
Proteins (gr)	74	90.96 ± 4.35	116	88.37 ± 3.47	126	72.63 ± 2.62	110	75.59 ± 2.81
Caloric intake (kcal/day)	76	2187.47 ± 83.09	115	2132.61 ± 67.49	129	1829.33 ± 54.85	112	1818.38 ± 58.99
**Caloric Expenditure**								
BMR(kcal/day)	78	1459.12 ± 21.21	122	1407.67 ± 16.95	131	1182.48 ± 9.24	126	1178.70 ± 9.43

PA (kcal/day)	52	543.59 ± 32.01*	43	389.50 ± 35.21*	107	307.84 ± 14.08*	38	241.68 ± 23.70*
BMR + PA (kcal/day)	52	2006.35 ± 56.70*	42	1786.87 ± 63.12*	107	1483.80 ± 21.84	38	1404.23 ± 36.77

Note: BC = Body composition; BMI = Body Mass Index; WHR = Waist-Hip Ratio; FMI = Fat Mass Index; FFMI = Fat Free Mass Index; SJ = Squat Jump; CMJ = Counter Movement Jump; ABA = Abalakov Jump; VO_2max = maximal oxygen consumption; BSSR = Back-saber sit and reach; SED = Sedentary; PA = Physical Activity; MVPA = Moderate-to-Vigorous PA; BMR = Basal Metabolic Rate. [1]Handgrip and BSSR are expressed as mean of right and left side. *Significant differences between rural vs. urban areas ($p<0.05$).
